# Overcoming the challenges facing Nepal’s health system during federalisation: an analysis of health system building blocks

**DOI:** 10.1186/s12961-023-01033-2

**Published:** 2023-11-02

**Authors:** Sharada Prasad Wasti, Edwin van Teijlingen, Simon Rushton, Madhusudan Subedi, Padam Simkhada, Julie Balen, for the Nepal Federal Health System Team, S. R. Adhikari, P. Adhikary, J. Balen, B. Bajracharya, S. Bhandari, S. Bhattarai, S. Gautam, A. Karki, J. K. Karki, B. Koirala, A. C. K. Lee, S. B. Marahatta, R. Neupane, S. Panday, U. Paudel, G. Pohl, S. Rushton, S. Sapkota, P. P. Simkhada, M. Subedi, E. van Teijlingen, A. Vaidya, S. P. Wasti

**Affiliations:** 1 School of Human and Health Sciences, University of Huddersfield, Huddersfield, United Kingdom; 2 School of Human Sciences, University of Greenwich, Greenwich, United Kingdom; 3 CMWH, Bournemouth University, Bournemouth, United Kingdom; 4 Department of Politics and International Relations, University of Sheffield, Sheffield, United Kingdom; 5 Manmohan Memorial Institute of Health Sciences, Kathmandu, Nepal; 6 School of Public Health, Patan Academy of Health Sciences, Lalitpur, Nepal; 7 School of Health and Related Research, University of Sheffield, Sheffield, United Kingdom; 8 School of Allied and Public Health Professions, Canterbury Christ Church University, Kent, United Kingdom

**Keywords:** Federalism, Health system, Qualitative research, Nepal

## Abstract

**Introduction:**

Nepal’s move to a federal system was a major constitutional and political change, with significant devolution of power and resources from the central government to seven newly created provinces and 753 local governments. Nepal’s health system is in the process of adapting to federalism, which is a challenging, yet potentially rewarding, task. This research is a part of broader study that aims to explore the opportunities and challenges facing Nepal’s health system as it adapts to federalisation.

**Methods:**

This exploratory qualitative study was conducted across the three tiers of government (federal, provincial, and local) in Nepal. We employed two methods: key informant interviews and participatory policy analysis workshops, to offer an in-depth understanding of stakeholders’ practical learnings, experiences, and opinions. Participants included policymakers, health service providers, local elected members, and other local stakeholders. All interviews were audio-recorded, transcribed, translated into English, and analysed thematically using the six WHO (World Health Organization) health system building blocks as a theoretical framework.

**Results:**

Participants noted both opportunities and challenges around each building block. Identified opportunities were: (a) tailored local health policies and plans, (b) improved health governance at the municipality level, (c) improved health infrastructure and service capacity, (d) improved outreach services, (e) increased resources (health budgets, staffing, and supplies), and (f) improved real-time data reporting from health facilities. At the same time, several challenges were identified including: (a) poor coordination between the tiers of government, (b) delayed release of funds, (c) maldistribution of staff, (d) problems over procurement, and (e) limited monitoring and supervision of the quality of service delivery and data reporting.

**Conclusion:**

Our findings suggest that since federalisation, Nepal’s health system performance is improving, although much remains to be accomplished. For Nepal to succeed in its federalisation process, understanding the challenges and opportunities is vital to improving each level of the health system in terms of (a) leadership and governance, (b) service delivery, (c) health financing, (d) health workforce, (e) access to essential medicines and technologies and (f) health information system.

## Background

An effective and efficient health system is required for achieving health improvement and meeting the Sustainable Development Goals (SDGs), in which governments pledged to “ensure healthy lives and promote well-being for all at all ages” [1]. A well-structured health system is crucial to meeting the needs of the population and also ensuring no one is left behind in striving towards health equity [1]. The decentralisation of health systems has been promoted as one way to improve health service delivery and has been central to health system reform in many countries [2]. The central tenets of decentralisation are shifting the authority to plan, manage and make decisions from the central government to lower levels, in theory bringing decision-making closer to the people, improving access to services, and empowering local government to improve the quality of services [3–5]. In Nepal’s case, health system decentralisation came about as part of a broader constitutional shift to a federal government system.

There is no universal definition of federalism, although all definitions involve more than one level of government (usually a central national government and regional provinces or states) dividing power, resources and responsibilities for the governance of a territory [6]. Here, we use the term ‘federalisation’ to denote the process of transitioning from a unitary to a federal governance system: a process that in Nepal (as in other countries that have undergone similar transitions) will take many years. Nepal’s process of federalisation (described further below) has been seen as both partial decentralisation and quasi-federalism [7]. Its introduction raised hopes that it could bring improvements to government service delivery, greater involvement of local people, increased resources, and better planning - including in the health system [8].

Existing evidence is mixed on whether either decentralisation or federalism leads to better health system performance; this depends on the context and implementation as much as on its governance architecture [5, 9]. In the case of Nepal, the federal government continues to play an important role in setting the basic parameters of health policy [10, 11]. A recent study indicated that federalisation has made the health system more flexible and responsive in adjusting to the changing needs of communities [12], although a lack of resources, conflicting policies across levels, poor leadership and weak implementation mechanisms [12, 13] have been identified as key issues impeding the provision of quality services [14]. This article examines the opportunities and challenges of Nepal’s federalisation process from the perspective of those holding a wide variety of health system roles, including policymakers, health service providers, local elected members, and other local stakeholders.

### Health system devolution in Nepal

Until 1974, several health policies were implemented by the Government of Nepal on an ad hoc basis. From 1975 to 1990, Nepal’s First Long-Term Health Plan was implemented, gradually expanding primary healthcare services to rural areas [15]. This expansion gained momentum after 1990, with the creation of a nationwide local health service structure at the ‘Village Development Committee level’. This included elements of decentralisation, with the transfer of authority for planning, budgeting, and managing health services to local levels to enhance community participation, local ownership, and service delivery efficiency [16, 17]. A 20-year health plan (1997–2017) was the next major milestone in improving Nepal’s health services and setting long-term health goals, including the Millennium Development Goals (MDG) [18, 19]. During this period, Nepal also experienced a decade-long civil conflict (1996–2007) [14].

In 2004, the Government of Nepal initiated a comprehensive Health Sector Reform Program focusing on governance, service delivery, health financing, and human resource development [20]. In 2007, the Interim Constitution of Nepal agreed at the end of the civil war specified “free basic health care” as a fundamental right and the country introduced a policy to foster access to “Essential Health Care Services” (EHCS) for every citizen [21].

In 2015, the new Constitution of Nepal restructured the political system into a federal republic comprising three autonomous governance levels: federal, provincial, and local/municipal. This constitution declared that health is a fundamental human right, that every citizen shall have the right to free basic health services, and that no one shall be deprived of emergency health care [22, 23]. The federal government is still responsible for overall health policy formulation, providing the budget, developing national standards and regulatory frameworks, and the delivery of specialised healthcare through national hospitals. The seven provincial governments have responsibility for the delivery of basic hospital services and coordinating with both the local and federal governments. The 753 local governments (rural and urban municipalities) are responsible for the provision of primary health services [19, 22, 23]. It was hoped that these changes could contribute to making the health system more responsive to local needs and more accountable to citizens.

In practice, however, the shift from a highly centralised health system to a more decentralised model under federalism has faced both structural and operational issues including infrastructural weaknesses, a shortage of skilled staff, health worker absenteeism, poor accountability, delays in procurement and a lack of coordination [8, 24–30]. In addition, there has been confusion about the mandates, roles and responsibilities of the different levels of governance, and the lines of reporting between them [8, 25, 27]. However, no previous study has systematically explored key health system stakeholders’ perspectives on the opportunities and challenges for the health system resulting from federalisation.

## Methods

### Study design

We employed two qualitative methods: interviews and participatory policy analysis (PPA) workshops. Both approaches were used to provide an in-depth understanding of the practical learnings, experiences and opinions of key health system stakeholders from different levels of the system, examining what impact federalisation has had on their working lives, and what challenges and opportunities for the health system have resulted from it [31]. Participatory qualitative research is most appropriate for exploring such complex research issues [32, 33]. The consolidated criteria for reporting qualitative research (COREQ) guidelines were followed [34].

### Study sites and participants

The study was conducted with stakeholders at three levels: the federal government, three (out of seven) provinces, and nine (out of 753) municipalities. The sites were purposively selected to include provinces (Bagmati, Lumbini and Karnali) representing the three ecological belts of Nepal: Mountain, Terai and Hill, respectively and to cover both urban and rural areas. Bagmati Province is located in the central part of the country and is Nepal’s most populous province by area [35], including the Kathmandu Valley which is by far the country’s largest urban area. Lumbini Province is located in the Terai lowland and is also a famous pilgrimage destination for Buddhists worldwide. Karnali Province, in the Far West, is much more remote, with a highly scattered population, hard-to-reach areas, and high levels of poverty.

Health services and health outcomes vary widely across the three provinces. In the Kathmandu Valley, high-quality private healthcare services are available (for those who can afford to use them), in addition to government-run services. In Lumbini, primary healthcare facilities in each ward (the smallest political unit, below municipalities) are complemented by 20 government-run hospitals [36]. In Karnali, meanwhile, around 28% of the municipality’s wards do not have health facilities [37]. Above fourth-fifths of women (79.1%) in Karnali, 86.9% in Lumbini and 88.8% in Bagmati received the recommended antenatal care whereas the national average was 80.5% [38]. Teenage pregnancy is highest in Karnali Province (21%), followed by Lumbini (10%), and lowest in Bagmati (8%) [38]. The prevalence of delivery in a health facility with skilled providers is low in Karnali Province (59.9%) followed by Lumbini (76.4%) and Bagmati (80%) respectively [39]. Catastrophic household health expenditure was highest in Karnali (a reported 13.3%), followed by Lumbini at 11.1%, and 10.7% in Bagmati [40].

At the local level, we selected nine municipalities from these three provinces representing both rural and urban municipalities and which have a similar level of health service access: each selected rural municipality has at least one health post/primary health centre; each selected urban municipality has a district-level hospital. If there was more than one health post in a selected municipality, one health facility was randomly selected.

Study participants were purposively selected [33] from all three levels of government, including a range of roles to gain in-depth insights from diverse perspectives. A total of 145 participants including policy-level representatives (*n* = 49), health service providers (*n* = 48), elected members (*n* = 22), Female Community Health Volunteers (FCHVs;  *n *= 16) and other stakeholders (*n* = 10) were recruited for the key informant interviews representing the federal government, three provinces and nine municipalities. A total of 12 participatory policy analysis workshops were held in three provinces and nine municipalities, involving a total of 163 participants representing policy level (*n* = 61), health service providers (*n* = 56), elected members (*n* = 28) and other stakeholders (*n* = 18).

### Data collection

Data collection began in 2021. Interviews and workshops were conducted/facilitated by trained field researchers. An interview guide, consisting of a list of relevant topics and corresponding open-ended questions, was developed based on a review of policies and literature. The research team has diverse backgrounds and reviewed and refined the guides before the final interviews and workshops. Participants were recruited using both purposive and snowball sampling approaches. We prepared a list of potential participants (i.e. professional roles) who were knowledgeable and/or working in the health system. Interviews were conducted using the interview guides, mainly in-person. Nine were conducted virtually due to the COVID-19 travel restrictions. Interview guides were pre-tested [41] at all three levels, resulting in changes to the sequence of questions for the final interview guides. PPA workshops were organised with the cooperation of municipality/province health coordinators and facilitated by our research team. The workshops included a variety of participatory exercises and discussion formats. Six trained field researchers experience conducted the interviews and facilitated the PPA workshops.
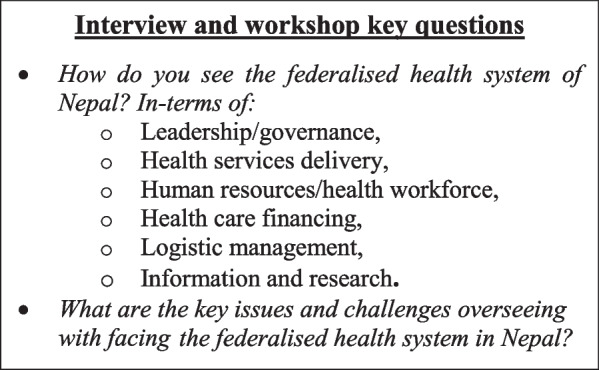


### Data management and analysis

All digitally recorded interviews were transcribed in Nepali and then translated into English. The translation was performed by trained Nepali and English speaking researchers who had extensive qualitative research experience. Other Nepali-speaking members of the research team independently reviewed the transcriptions and translations. Any disagreements were discussed to establish appropriate translation. Each transcript was reviewed at least twice while listening to the audio recording to ensure the accuracy of transcription and to improve familiarity with the data. All the transcripts were thoroughly reviewed and finalised by the core research team who were involved in the data collection. All transcripts were then imported into NVivo v20 software for coding and analysis.

**Fig. 1:**
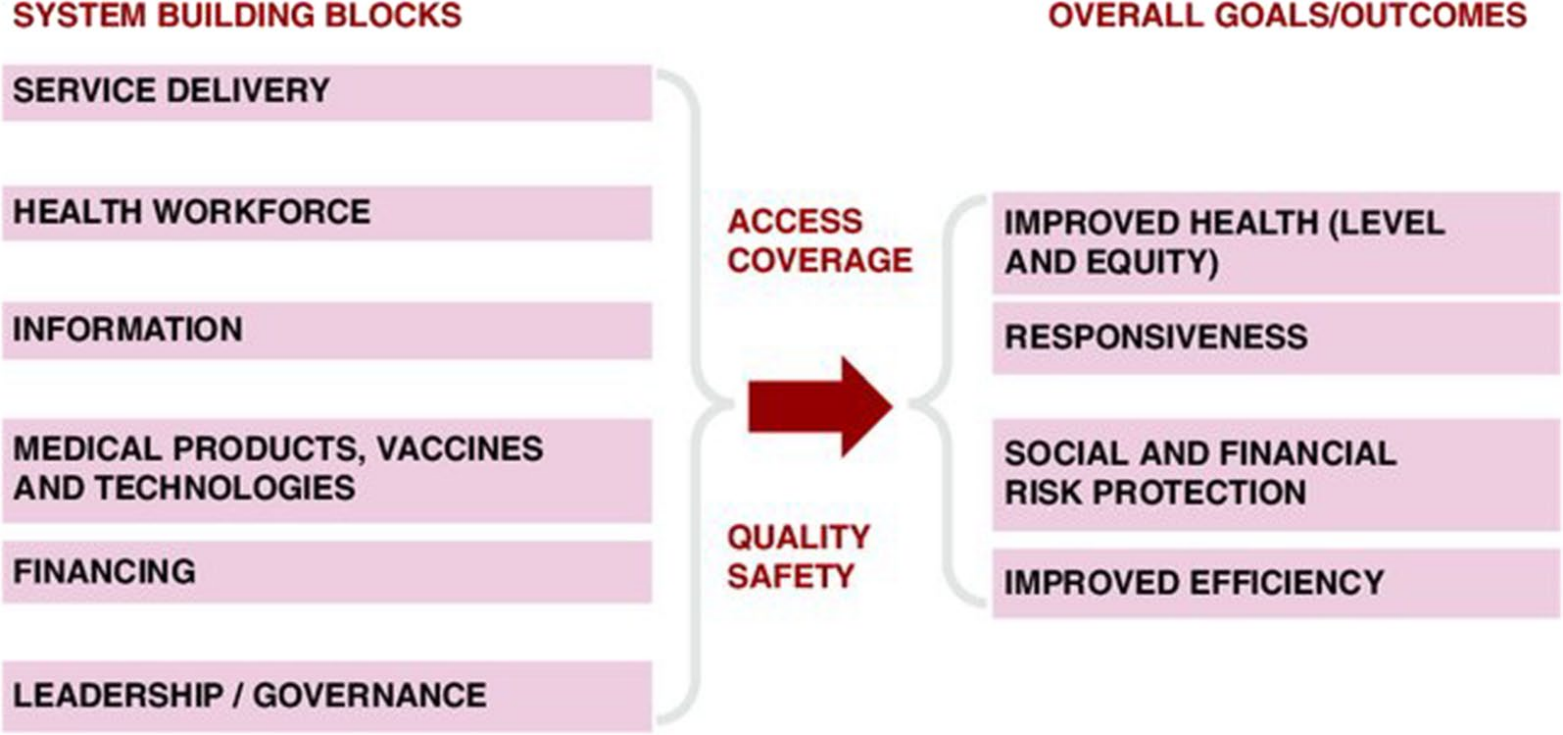
WHO health systems framework [42]

Data coding followed pre-determined themes based on the six WHO health system “building blocks” framework [42]. The building blocks (Fig. 1) are typically used to assess the system holistically, providing a common language and reference point for researchers and policymakers [42–44]. The quotes presented best represented the range of ideas voiced around key themes.

### Ethical consideration

The study protocol was approved by the Institutional Review Board of the University of Sheffield, the UK and the Nepal Health Research Council (Reference No.: 1030/2020). All participants signed an informed consent form before being enrolled in the study. Confidentiality was maintained throughout data collection, analysis and publication.

## Results

**Fig. 2:**
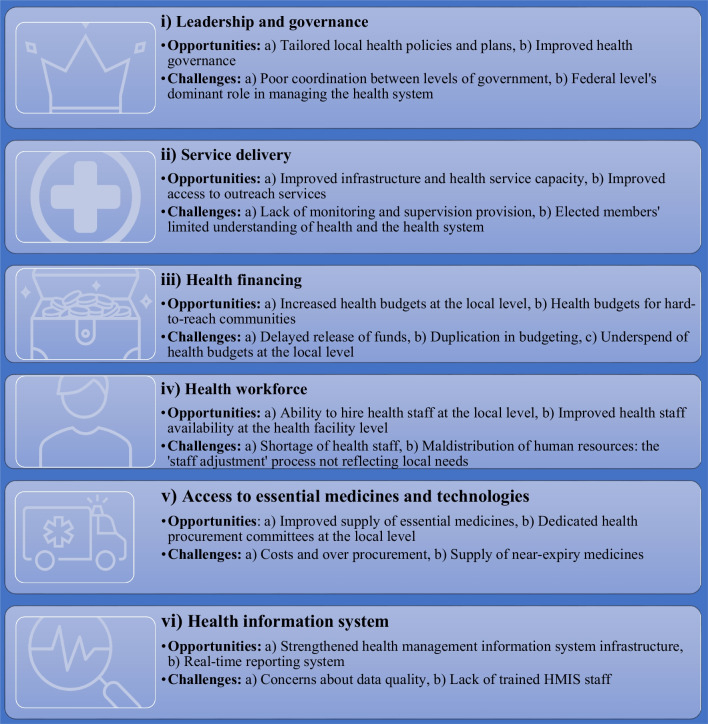
Opportunities and challenges facing the federalised health system in Nepal

The key findings of the study are summarised based on the WHO’s six health system building blocks. The categories for each theme included both opportunities and challenges identified by the study participants (Fig. 2).

### Opportunities and challenges in Nepal’s federalised health system

Each section below begins with summarising stakeholders’ perceptions of the opportunities followed by challenges in the respective health system building blocks:

i) Leadership and governance
  - Tailored local health policies and plans
    The new system has produced a large number of health policy-related documents (acts, strategies, plans and guidelines). Participants repeatedly stated that local governments now have independent power and authority to design and implement policies and plans according to their local needs. Some local governments had produced their own health policies. Local-level elected members saw the dramatically increased number of policy-related documents as an advantage:
    *While working we have identified the needs and have been formulating many plans, policies, rules, and regulations accordingly. In the past, we had no [local] plans, policies, rules and regulations, but now we have around 80-90 rules and regulations. So, while working we can identify problems and needs as well (P-16, Sindhupalchok-M-Elected Member)*
  - Improved health governance
    Local leadership and accountability also improved, with a dedicated Health Coordinator in each municipality who works both at the health facility level and in coordinating with the provincial government and other line agencies. Participants repeatedly stated that the provincial-level health leadership has also increased and taken the lead role in managing health facilities.
    *I think now health leadership has gradually increased and been delegated to the provinces and local levels. Specialized hospitals will be set up in every province with the leadership of provinces and each province will be gradually increased under the leadership of the provinces and health services in each province (P-5, Federal, MoHP Official)*
    Participants further mentioned proactive-local leadership in the federal structure:
    *We have seen the proactive role and responsibility of the three levels of government. Each level of government has initiated and played a crucial leadership role. We have seen that the federalisation of health at the local government level has increased accountability and responsibility (P-9, Federal-Policy Researcher)*

The following challenges were identified under the leadership and governance building block:

- Poor coordination between levels of government
  The lack of coordination between the three tiers of government was strongly echoed by participants at all levels. The main concerns were the disconnection between the Federal Ministry of Health and Population (MoHP) and the health facility level. Almost all health worker participants were worried that the new system made it more difficult to manage day-to-day health activities because of the loss of District Health Office leadership, which had previously been the key link between the central government and health facilities. Due to limited coordination, a number of issues of duplication of programs have arisen.
  *There is no proper chain of command between different tiers of government. There has been duplication in so many training-related programs due to the lack of coordination. We have been invited to take part in the same training from the federal as well as provincial levels at different times. If there is good coordination, then resources could have been saved and could be used for other purposes (PPA-Mugu District)*
- Federal level’s dominant role in managing the health system
  Many participants felt that the system was not working as the new Constitution intended as the federal MoHP was continuing to play a dominant role due to a centralised mindset.
  *Still, the federal level authority thinks that they should not provide the power to the lower levels, because the local level lacks the capacity to perform their tasks. The local level can perform the activities, but due to the mentality of the central level they have not been able to perform them (P-23, Kathmandu, Health Service Providers)*
  Provincial policy-making participants agreed that the federal government was playing a dominant role in managing the health system due to frequent staff changes at the provincial level.
  *Looking from a bureaucratic perspective, there is a dominant role of the federal level government and the health staff think that the central level [MoHP] should mobilise all staff, like in the unitary system. It’s been 2.5 years since we have been working here, and during this period 9/10 Ministry of Social Development health secretaries have been changed (P-8, Karnali Province, Policy Level)*

ii) Service delivery
  - Improved infrastructure and health service capacity
    The consensus was that the number of health facilities has increased after federalisation. The current system enables the establishment of new health facilities, especially since the decision to do so rests at the local government level, which was not possible in the previous unitary health system. There have been significant achievements in establishing new health facilities such as health posts and birthing centres in the community and the upgrading of existing facilities.
    *New health facilities have increased vastly. We had few health facilities in earlier days but now we have two hospitals and 125-126 health posts in our district. The current local level government role and responsibility has increased the number of health facilities at the community level (P-2, Sindhupalchok, Stakeholder)*
    There were not only new health facilities, but interviewees also noted the improvement in the capacity of the pre-existing ones.
    *One good aspect of federalisation is that we have upgraded 15-25 bed hospitals into 50-bed hospitals. We are establishing many other hospitals as well. We have acquired almost all the medical equipment necessary for diagnosis (P-4, Lumbini, Health Service Provider)*
  - Improved access to outreach services
    Participants repeatedly reported that the current system has greater provision for outreach services to those who are unable to come to health facilities like the elderly, people with disabilities, pregnant mothers and newborns.
    *Now there’s almost one health post in each ward so people are getting our services efficiently and for those who are not able to come, we reach out to them by visiting their homes (P-12, Sindhupalchok, M-FCHV)*
    *I think after federalisation, people who are from vulnerable groups like people with disabilities, elderly people, pregnant women, and children have been getting services in their homes, which was not the case under the previous health system (PPA-Mugu District)*
    Participants also reported that the federalised system had taught the public about the importance of health and health-seeking behaviours. The latter was reported to have changed significantly, even in remote communities that used to use traditional healers before modern health services.
    *Before federalism, people had to walk for 2–3 hours to get Cetamol [brand name for paracetamol in Nepal]. That was the reason for the early death of some elderly people. People used to have medicinal herbs and use home remedies for treatment as they had to walk a long distance to get medicine. Slowly people are understanding that instead of … using medicinal herbs or home remedies, they have to visit the health post for treatment (P-10, Mugu, RM-HP In-Charge)*
    Almost all agreed that the federalised health system has improved services for both general populations as well as marginalised communities, with local governments having health as the top priority.

Key challenges were identified under Health Service Provision:

- Lack of monitoring and supervision provision
  Health service providers in particular raised concerns that there is little monitoring and supervision as compared to the days when District Health Offices were responsible for monitoring and mentoring health service delivery staff. This was reducing the opportunity for feedback that could support service improvement.
  *Now after federalisation, no one visits from the district level after the responsibility has been moved to the local level. It has been difficult to meet our focal person and we are confused about who we should share our queries with. Before federalisation, the focal person used to visit and I find that’s the difference after federalisation (P-14, Nawalparasi West, M- Health Service Provider)*
- Elected members’ limited understanding of health
  Levels of knowledge and understanding regarding health and health programs amongst local-level elected officials were thought to be highly variable. Participants felt that this could impede planning and proper prioritisation of programs. Some participants felt that this often led to a narrow focus on curative services rather than prevention.
  *Local elected representatives are not technically sound, so they tend to overlook some issues. Most of the time, it is difficult for us to make them understand the need to prioritise the health sector. Elected representatives could not understand health and had difficulty understanding the existing health policies (PPA-Mugu District).*

iii) Health financing
  - Increased health budgets at the local level
    Participants from both urban and rural municipalities appreciated and acknowledged that the federalised health system has allocated health budgets more fairly than the unitary government, where it was felt that power and political considerations influenced budgeting to certain districts and municipalities. Likewise, the size of budgets had also increased.
    *Compared to before, there is more budget available after the implementation of federalisation. Before federalisation, only 15-20 lakhs [around $12,000-$16,000] were provided, and now one-two hundred thousand [around $78,000-$156,000] is available at the ward level. If this budget is utilised efficiently then the health sector could be well-developed (P-5, Nawalparasi West-M-Admin Staff)*
    Policy-level participants also agreed about the increased health budgets after federalisation.
    *The first thing is the volume of budget that goes to the local level has increased. The health activities at the local level have been increased. Previously, the budget used to get allocated based on power or good relationships (P-16, Federal-Policy Level)*
b) Health budgets for hard-to-reach communities
  Participants at all three levels repeatedly said that under the federal system, the government has for the first-time prioritised health budgets for difficult-to-reach communities, especially for maternal and neonatal health and nutrition, and services for older adults, people with disabilities, and cancer patients—particularly those from marginalised or deprived communities. Due to the devolution of power and resources, each local government has planned and implemented programs as per the local needs, targeting local priorities such as strengthening birthing centres, introducing free ambulance services, or launching school health programs:
  *After federalisation.... all the responsibilities have been provided to the municipality level. Responsibility to help and support the elderly and marginalized populations. Recently I have seen a ward distributing warm blankets to these vulnerable populations. In my opinion, it has been improved in comparison to the past. It is because such responsibilities have been included in the activities and programs at the municipality level. There is a certain budget allocated for poor, marginalized and vulnerable populations which has been a bit higher than in the past (P-2, Lalitpur- Health Service Provider)*

Several challenges were identified under the health financing building block:

- Delayed release of funds
  Local stakeholders repeatedly reported that delays in getting funds impede program implementation.
  *We ask for the budget at the local level, they often reply we have not received it from the upper level [Federal or Provincial]. … I do not know if these funds have been provided to the rural municipality, but there are no such funds here at the health post level (P-1, Sindhupalchok, RM- Health Service Provider)*
- Duplication in budgeting
  Local-level study participants raised the concern that there are issues at the local level caused by different tiers of government that had budgeted for the same activity.
  *The federal level has allocated different programmes at different levels. Some of them have been duplicated programmes, but we conducted those duplicated programmes at different venues (P-2, Lumbini Province, Policy Level)*
  PPA participants also pointed to the duplication of health promotion materials, whereas under the unitary system, all such procurement had been done centrally.
  *We have the problem of duplication in the printing of materials related to awareness-raising activities. Previously, only the central level was used to print and distribute those materials (PPA-Bagmati Province)*
- Underspend of health budgets at the local level
  Although each municipality has been allocated a budget for health, participants raised concerns that the money had not always been spent well—or that money had gone unused.
  *In Karnali province, in the past two years, only 20% of the budget has been used by the [Province] government and the remainder has been frozen. In the first year, Karnali province used their budget to purchase vehicles/cars and in the second year also only 20% of the budget has been used. So, we are in a critical situation for annual budget use (P-8, Karnali Province- Policy Level)*

iv) Health workforce
  - Ability to hire health staff at the local level
    Participants highly appreciated the new possibility to hire short-term or temporary staff at the local level, using local budgets, to address personnel shortages. Participants further highlighted that hiring and deploying local staff improves services because they know the community and their health needs:
    *After federalism, I feel there have been many positive changes in the health sector. The reason I feel it is staff management, which has been handed over to the local government under the Conditional Grant (Sasakta Anudan) from the Federal Government Budget. This allows the local government to hire employees temporarily (P-24, Sindhupalchok, RM-Elected Member).*
  - Improved health staff availability at the health facility level
    Participants gave credit to the federal system for improvements in local health staff availability.
    *The timings of staff attendance in the health facilities were not regular; they attended their office any time when they wanted under the unitary system. This has become mandatory and has improved vastly, with staff on duty from 10 AM to 5 PM after federalisation (P-5, Sindhupalchok-RM- Health Service Provider)*
    Participants further said that federalism had fostered improved accountability.
    *After federalisation, things have improved. Health staff have better understood what their role and responsibility is. They didn’t understand before- that is one reason I think federalisation has been implemented now (P- 20, Kathmandu, M- Health Service Provider)*

Key challenges were identified under the health workforce building block:

- Shortage of health staff
  Health staff shortage was one of the most commonly discussed issues among the local-level participants. The shortage of health staff, they felt, directly impeded the quality of health services and meant that staff were overwhelmed by their daily work. In one of the remote provinces, policy-level participants reported that the shortage of trained health staff has hugely obstructed the quality of health services.
  *Service delivery is highly impacted by a scarcity of manpower and almost all the indicators have declined. The workload has increased. 1/3rd of positions are filled, and 2/3rds are still vacant in the case of clinical doctors, 2/3rds are filled and 1/3rd are vacant in the case of paramedics and administrative staff (PPA-Karnali Province)*
- Staff adjustment not reflecting local needs
  After the federalisation of the country, the government reallocated government employees over the three tiers; a long process that took place in all sectors, including health [30]. Reallocation was based on staff’s place of birth, whilst senior-level staff were deputed at the federal and provincial levels and junior (functional level) staff were put at the local levels. Most participants noted problems with this staff adjustment system, which had resulted in staff not being in the right post. Although some health facilities have an adequate number of staff, they do not necessarily have the right mix of skills—and existing staff skills were not always being fully utilised:
  *The major problem here was raised during the adjustment of the health staff. I am talking from the aspect of nurses, one of our nurses who was trained as an Anaesthesia assistant is now sitting at a Health Post in a Rural Municipality. She is not able to use her skills properly. On the one hand, she is frustrated. On the other hand, her skills are not being utilized. (P-9, Federal, Policy Level)*

v) Access to essential medicines and technologies
  - Improved supply of essential medicines
    A large number of participants perceived that, after the federalisation of the country, medicine supply and procurement has been better than under the unitary structure, when the supply system was centralised. Participants repeatedly mentioned that essential medicines and other health commodities are being more regularly supplied:
    *Before federalisation, I am not sure how the district level used to provide the necessary medical supplies and other stuff. But now as we are under the rural municipality, based on our needs we have been receiving all the essential supplies from them (P-5, Nawalparasi West, M- Health Service Provider)*
    In addition, the Logistic Management Information System (LMIS) has gradually been shifted into the Electronic Logistic Management Information System (eLMIS) for real-time reporting, which helps with regular monitoring of stock status to prevent stock-outs at the facility level.
    *After federalism, much work has been done in the context of information. Now we are slowly expanding the eLMIS system. eLMIS training has also been rolled out and many staff have already been trained. The long-term vision is to make the health facilities from where data is generated online and in real-time (P-11, Federal, Policy Level)*
  - Dedicated health procurement committee at the local level
    The federalised health system has resulted in the formation of procurement committees at the local level that help ensure the increased availability of essential medicines. Each municipality and the provincial government has a dedicated procurement unit that regularly looks at the logistic status and stock levels at local-level health facilities. This has improved the availability of medicines and other supplies, reduced stockouts, and improved the government’s accountability to the citizens. The current procurement and supply practices are seen as very transparent to citizens compared to the unitary system.
    *We have a procurement committee, and we plan procurement as per the requirement of medicines and accordingly procure it from the municipality health office (P-12, Sindhupalchok, Elected Member)*

Various challenges were identified under the access to essential medicines and technologies building block:

- Costs and over procurement
  Policy-level participants repeatedly stated that procuring medical items from multiple channels increased costs enormously. Participants also reported that there was over-procurement of some items from the local level. For example:
  *If the medicines were centrally distributed as they were under the previous system, the cost of medicines would be cheaper. For example, medicines that used to cost one rupee in the previous system, now cost seven rupees. The cost of health services has significantly increased (PPA-Lalitpur District)*
  *After federalism, all levels of government can procure medicines. We have also seen an overlap in procuring through multiple doors. Although we have lists of medicines procured from different levels of government, we repeatedly hear that there is duplication of the procurement. Some medicines are brought from federal, provincial, and local- some medicines are in surplus, and some are scarce. There is a need to rectify that [P-5, Federal—Policy Level)*
- Supply of near-expiry medicines
  Participants reported many instances in which health facilities had received near-expiry medicines. This issue was not only present in remote health facilities but even in capital city health facilities.
  *We even received medicines that were near the expiry date. We used them, but once they expired, we could not distribute them, so we did not. When we gave them the feedback that this way will not work, they said that the government system is changing slowly and gradually it will all be managed. The changes are not complete, so it is still kind of confusing for us as we only coordinate with Metropolitan (P-15, Kathmandu-District, Health Service Provider)*

vi) Health information system
  - Strengthened health management information system infrastructures
    Participants reported that health facilities’ online reporting capacity has been improved at local health facilities with expanding internet services, computer facilities with computer operators, and also the installation of the new health information and data management system [District Health Information Software (DHIS-2)] at local health facilities. The quality of the online recording system has been vastly improved, and a data quality control mechanism has also been addressed by setting data reporting deadlines.
    *Our online system capacity has been increased. We are doing all reporting online. Now we are using the DHIS-2 [District Health Information Software] tool for HMIS strengthening (P-4, Lumbini, Health Service Provider)*
  - Real-time reporting system
    The study found that regular and real-time reporting was in practice in the study municipalities. Before the federalisation of the country, HMIS relied on paper-based data recording, transported upwards from the health facilities to the higher levels. This delayed reporting and led to incompleteness or inconsistency of data. After federalism, the routine health data reporting system in some areas has moved to the DHIS-2 platform, which has enabled real-time reporting and means that data can be accessed more easily by stakeholders who have user rights to the system.
    *I think this is the first rural municipality in Sindhupalchok in which we have internet facilities in all our health posts. We have 11 health posts. Among them, eight health posts have an online reporting system. This is gradually improving and can get information across to the line ministries. HMIS is vastly improved (P-25, Sindhupalchok-RM-Official)*

The health information system building block also faced several challenges:

- Concerns about data quality
  Consistent and systematic compilation of data with regular analysis and interpretation would foster evidence-based decision-making and clinical practice, but participants raised concerns that the health information management system is poor and that no regular quality control measures are in place. Health facility staff agreed that there is no data review or data quality control mechanism.
  *We do not have technical staff dedicated to M&E [monitoring and evaluation], data review and verification. We do not have any new opportunities for data review and verification after federalisation. Previously, the focal person used to conduct the different health programs, conduct data quality assessments, and update the health information. But now, the update of health information is almost zero (P-3, Lalitpur, Health Service Provider)*
- Lack of trained HMIS staff
  Human resource management emerged as one of the major challenges, and the lack of trained staff was making it difficult to roll out the eHMIS system.
  *DHIS-2 [District Health Information System-2] has been rolled out. They have been prioritizing regular recording and reporting of health information under HMIS and LMIS [logistic management information system]. But we do not have dedicated trained staff. There is a lack of expertise for online reporting because of the lower-level staff at the health facility level (P-17, Nawalparasi West, RM- Stakeholder)*

## Discussion

Participants in the interviews and PPA workshops identified both opportunities and challenges concerning the six WHO health system “building blocks”. These insights provide credible information for decision-making to strengthen the health system in Nepal. This section discusses each of the six WHO health system building blocks in turn.

*Leadership and governance:* Our findings revealed a perception that health sector leadership has been gradually improving. A similar finding was revealed in a recent study which showed that local-level governance and management had vastly improved under the federal structure, which was evident in local government management of the COVID-19 response, including quarantine, testing and isolation [26]. The pandemic created both opportunities and challenges for the federal health system and the local-level government role was highly acknowledged and appreciated in managing the pandemic [13, 45]. Health governance is also improving at the provincial level [37]. The experience of devolution in Nepal is consistent with the idea that federalism brings decision-making closer to the community [7]. However, a recent National Health Facility Survey (2021) revealed that just above one-third (36%) of health facilities completed financial audits [46], which suggests the need for further improvements in governance to enable them to be held accountable.

The lack of coordination between and within tiers of government remains a major challenge in the current health system. The 2015 Constitution set in motion a major process of state restructuring, introducing new mechanisms for power-sharing, but the three tiers of government have also led to a degree of bureaucratisation, as well as confusion over respective roles [27]. Strengthening coordination between the three tiers of government would enhance more accountable and responsive governance.

The findings also provided evidence that some of the challenges are products of the fact that the system is still in transition: a number of new policies and processes have been initiated and need to be fully implemented; new governance and leadership roles and responsibilities need time to become fully functional [13]. Our findings are also concordant with the earlier study conducted by Adhikari (2020) in Nepal that federalism has faced several challenges such as the centralised mindset of leaders and bureaucrats, unitary organisational structure, lack of local level policy, institutional and legal framework, interruptions of service delivery and limited local level involvement in planning and budgeting processes [8, 13, 14, 25].

*Service delivery:* Our findings show a perception that health service coverage and access have increased, with more services being available closer to the community in some areas. Several health facilities have already been created or improved, as per the national health policy (2019)’s vision of ‘one municipality—one hospital’ [19, 30]. A recent study indicated that 79.8% of the population could access primary health facilities within 30 min walk, which is higher than the Nepal Living Standard Survey in 2010/11 (which was 61.8%) [47, 48]. However, our findings also raised concerns about the lack of monitoring from the new provincial governments that may impede the quality of services. This issue was also highlighted in another recent study [49] where the monitoring from the provincial government was reported to be happening on an ad-hoc basis. There is a clear need for provincial governments to provide more comprehensive monitoring and mentoring support at the local level.

Similarly, our findings imply that local governments have a limited level of understanding of health, although they have primary responsibility for local service delivery. As a result, there is a need for capacity building at the lower levels of government: a recent study, which aligns with our findings, indicated that local governments require additional support from the federal government to plan, budget, manage and monitor health programmes [50].

*Health financing:* Under the federal health system in Nepal, health has been prioritised—as seen in increasing health budgets. Budget allocation is evident from all three tiers of government. The volume of the health sector budget has significantly increased, from 4.6 per cent in 2017/18 to 8.6 per cent in 2021/22 [51]. Likewise, the Karnali Province budget rose from 5.3% in 2019/20 to 7.8% in 2020/21 [37] and Lumbini reached up to 10% [52]. It is noteworthy, however, that a large proportion of the increased budget has gone to infrastructure development and curative services rather than public health services.

Despite this, our findings revealed stakeholders’ concerns about budget duplication, and budgets not being released in a timely manner, which makes it difficult for local governments to spend the allocated health budgets within the fiscal year and smoothly manage programmes. Several studies from other countries have also indicated that a decentralised health system is associated with higher health expenditures and weaker mechanisms for resource management at the sub-national level [53–55]. Further studies into health budgeting are important to more clearly understand why health budgets are not being released on time, and whether there are other reasons for underspending at the local level.

*Human resources:* Effective workforce management is critical. Our findings indicate a mixed picture, with significant new opportunities for staff management at the provincial and local levels (for example, recruiting directly at the local level). Despite this, staff shortages and the maldistribution of skills were some of the most prominent issues in our study. The issue of human resources for health was problematic before federalisation, and it has not been resolved adequately yet [56–58]. Delays in the establishment of province-level Public Service Commissions are impeding the recruitment of permanent staff. Therefore, the three levels of government should closely work together and make and implement the necessary laws and policies as per provincial and local needs.

*Access to essential medicines and technologies:* One significant finding of this study is that federalisation has led to the establishment of strong procurement committees at the local level that oversee and are accountable for the supply of medical commodities. The recent Nepal Health Facility Survey (2021) findings indicated that 59% of health facilities have all basic amenities and the availability of basic medical equipment (41%), a sharp increase from 13% in 2015. All provinces in this study have a slightly higher rate of availability of medical equipment than the national average (54.3% in Lumbini, 45.7% in Karnali and 41.1% in Bagmati) [46, 59].

Other studies in Nepal have also shown that adopting the ‘pull system’ has improved the stock situation at the facility level, despite increasing demands for essential medicines [19]. Another reason for improving supplies may be the introduction of the electronic Logistic Management Information System, which generates information for demand forecasting, quantification and evidence-based procurement [19]. However, our findings also suggest that under the federal system, the purchase of medical supplies has become more expensive as a result of reductions in economies of scale [25]. It is noted that a total of 753 local municipalities procure commodities which need quality control mechanisms and standards. In addition to cost, despite logistic improvements, there is still a limited supply of essential medicines. A health facility survey’s 2021 findings showed that only 1.3% of health facilities had availability of all 18 trace medicines, only a very marginal improvement on the findings of the 2015 survey [46]. We also found participants complaining about the supply of near-expired medicines, which was also found in an earlier study [60]. Therefore, improved real-time monitoring of stock levels in both stores and health facilities is required.

*Health information system:* Vast improvements have been seen in HMIS infrastructure at local health facilities. Electronic reporting through the DHIS has been gradually expanded [30], and data shows that almost all (99%) of health facilities have been regularly reporting routine data electronically [30]. Seven out of 10 health facilities had trained DHIS-2 staff, although there was variation by province: Lumbini has a higher proportion (79.2%) than Bagmati (61.9%) and Karnali (63.1%) [36]. Despite this, our findings revealed that there is significant concern about the quality of this data. A recent MoHP review also indicated that despite improvements in routine data reporting, completeness, timeliness, and data quality issues are still challenging [30]. There is a need for further staff training to increase capacity and understanding of the importance of real-time reporting and data quality.

Overall, stakeholders already saw signs of improvement, not least concerning increased availability of a skilled health workforce, establishment and upgrading of health facilities, reduction of staff absenteeism, availability of essential medicines, increased health budgets, and access to health services at the community level [19, 30, 61]. People felt the presence of government at the local level, making relevant policies and plans as per local needs. The National Health Policy (2019) recognises the three tiers of government, which has significantly added the policy contents to strengthen the federalised health system compared to the unitary health policies [17, 18, 23]. Before federalisation in Nepal, health budgets were allocated only from the central government, but after the federalisation, budgets have been allocated from the three tiers of government and also increased after COVID-19 [13]. Many of the previously identified challenges of Nepal’s health system have been improved in the current federalised health system, although there is still room for further improvement. Addressing these six-health system-strengthening building blocks requires sustained efforts from the governments to enhance healthcare workforce distribution, increase funding, strengthen health infrastructures and information systems and prioritise the quality assurance mechanism.

### Strengths and limitations of the study

This study was the first of its kind after the federalisation of the country. The study ensured good representation from all three tiers of government and included both health and non-health (e.g. political) participants. This study has used both key informant interviews and participatory policy analysis workshop data representing policymakers, service providers, and local elected leaders from the three levels of government.

A key limitation was the collection of data during the early days of federalisation where the implementation of federalism has not even been completed one tenure (five years) of the newly elected governments. Therefore, this paper is described as looking at Nepal’s health system in transition but not the ‘final effects’ of federalisation. The current study only included three of Nepal’s seven provinces and a small selection of municipal governments. Therefore, the findings of this study may not resonate with other provinces and municipalities. This study did not include beneficiaries, particularly the general public/patients whose perceptions of improvements (or otherwise) in the health system may differ from those of professional health system stakeholders.

## Conclusion

Nepal’s move to a federal system has created opportunities for improving health services, but efforts to strengthen the health system are ongoing and much more needs to be done. The devolution of power and resources, the adoption of new health policies, and increasing resources (financial, health staff, supplies and health information system) were identified by participants as key opportunities to improve Nepal’s health system. However, the system is still in transition, and it may take time to capture the full benefits of federalisation. During the transition, there are some significant challenges, including a lack of coordination between and within governments, a lack of program monitoring and supervision, duplication of programs and funds, the untimely release of funds, underspent funds, staff shortages, maldistribution of skills, and continuing problems with medicine supply. All three tiers of the government should have close coordination to address functional level issues, as well as to implement measures to ensure adequate and appropriate staffing, reliable supply of commodities, effective budget management, and a monitoring system to ensure the quality of services being provided to every citizen. We recommend the setting up of a mixed-methods study to better understand beneficiaries' perceptions of the federalised health system and the effects of the system on access to and the utilisation of health services across the provinces in Nepal.

## Data Availability

The datasets are available from the corresponding author on reasonable request.

## Abbreviations

COREQ: Consolidated Criteria for Reporting Qualitative Research
COVID-19: Coronavirus disease
DHIS-2: District Health Information Software
eLMIS: Electronic Logistic Management Information System
FCHVs: Female Community Health Volunteers
HMIS: Health Management Information System
LMIS: Logistic Management Information System
M&E: Monitoring and Evaluation
MDG: Millennium Development Goal
MoHP: Ministry of Health and Population
PPA: Participatory Policy Analysis
SDG: Sustainable Development Goal
UK: United Kingdom
WHO: World Health Organization
